# Significant Association of Estrogen Receptor-β Isoforms and Coactivators in Breast Cancer Subtypes

**DOI:** 10.3390/cimb45030166

**Published:** 2023-03-17

**Authors:** Young Choi, Simcha Pollack

**Affiliations:** 1 Department of Pathology, Yale School of Medicine, 434 Pine Grove Lane, Hartsdale, NY 10530, USA; 2 Department of Statistics, St. John’s University, New York, NY 11423, USA

**Keywords:** estrogen receptor β, coactivator, correlation, coregulation, prognosis, therapy

## Abstract

Nuclear receptor coregulators are the principal regulators of Estrogen Receptor (ER)-mediated transcription. ERβ, an ER subtype first identified in 1996, is associated with poor outcomes in breast cancer (BCa) subtypes, and the coexpression of the ERβ1 isoform and AIB-1 and TIF-2 coactivators in BCa-associated myofibroblasts is associated with high-grade BCa. We aimed to identify the specific coactivators that are involved in the progression of ERβ-expressing BCa. ERβ isoforms, coactivators, and prognostic markers were tested using standard immunohistochemistry. AIB-1, TIF-2, NF-kB, p-c-Jun, and/or cyclin D1 were differentially correlated with ERβ isoform expression in the BCa subtypes and subgroups. The coexpression of the ERβ5 and/or ERβ1 isoforms and the coactivators were found to be correlated with a high expression of P53, Ki-67, and Her2/neu and large-sized and/or high-grade tumors in BCa. Our study supports the notion that ERβ isoforms and coactivators seemingly coregulate the proliferation and progression of BCa and may provide insight into the potential therapeutic uses of the coactivators in BCa.

## 1. Introduction

### 1.1. Two Estrogen Receptors

There are two estrogen receptor (ER) genes (ESR1/ERα and ESR2/ERβ). ERα and ERβ are members of the nuclear receptor superfamily of transcription factors and share some structural similarities, including a high degree of homology (96%) in their DNA-binding regions. However, they also have distinct differences in genotype, tissue distribution, and binding to pharmacological agents; they share only moderate homology in the ligand-binding region, and they have markedly distinct NH^2^-terminal activation function-1 (AP-1) regions. ERα and ERβ can form heterodimers [1]; when coexpressed, ERβ acts as a transdominant inhibitor of ERα transcriptional activity. Thus, the relative levels of ERα and ERβ in breast cancer (BCa) are likely to affect cell proliferation, signaling pathways, and their response to ER ligands [2,3]. ERβ has different variant forms that interact with multiple protein partners, as well as ligands, and heterodimerize with ERα, thereby creating a highly complex labyrinth of functions. Furthermore, ERβ localizes in different cellular compartments and is susceptible to different posttranscriptional modifications (PTM) [4,5,6].

The exact role of ERβ in BCa has not yet been fully established. Highly variable and even opposite effects have been ascribed to the expression of ERβ isoform mRNA and protein expression in BCa, including both proliferative and growth-inhibitory actions, as well as favorable or adverse clinical outcomes [7,8]. Our recent study showed that ERβ1 protein expression is associated with poor prognostic markers [9]. ERβ2 and ERβ5mRNA expression are risk factors for OS in BCa subtypes and are associated with poor prognostic biomarkers, particularly in ERα-negative BCa and TNBC [10]. Overall, the outcome results of ERβ expression in BCa are inconsistent. Such inconsistent and controversial results may be due to the complexity of ERβ isoforms and the lack of standardized testing protocols but may also relate to various downstream signaling pathways, their PTM, and the their involvement of coregulators in its transcription.

### 1.2. Nuclear Receptor Coregulators

Nuclear receptor (NR) coregulators have emerged as the principal regulators of gene expression by directly interacting with and modulating the activity of NRs. ER-mediated transcriptional and biological activities require the recruitment of a diverse array of coregulator proteins to ERs. Coregulator complexes enable the ERs to respond to hormones or pharmacological ligands and communicate with the transcription apparatus at target gene promoters. Ligand-dependent and ligand-independent ERα and ERβ receptors recruit coactivators and corepressors and activate or repress ER-mediated transcription [11,12,13,14,15]. Alterations in ER conformation induced by binding to different estrogen response element (ERE) sequences modulate ERα and ERβ interaction with coactivators and corepressors [16]. 

Steroid receptor coactivator (SRC) family members, the p160 class, of coactivators are a gene family characterized as the primary coactivators for NRs and are required for NR-mediated transcription. They have been widely implicated in the regulation of steroid hormone action by mediating functions of NRs and facilitating the assembly of transcriptome complexes at target genes [14,17,18]. The SRC family consists of three members: SRC-1 (NCOA1), transcriptional intermediary factor-2 (TIF-2/SRC-2/GRIP-1/NCOA2), and amplified in breast cancer-1 (SAIB-1/SRC-3/NCOA3). The alteration or deregulation of SRC coregulators is common in BCa and enhances both ligand-independent and ligand-dependent ERα signaling to drive the proliferation, progression, and invasive capacity of neoplastic cells [13,14,15,19].

Among the SRC family members, SRC-3/AIB-1 is the primary coactivator for ERα and is overexpressed in BCa, and it is a crucial driver of mammary tumorigenesis [20,21,22,23,24]. AIB-1 mRNA and protein overexpression correlate with the expression of high Her2/neu, larger tumor size, higher tumor grade, and poor disease-free survival (DFS). AIB-1 also interacts with coactivates p65/NF-κB and plays an essential role in the NF-kB signaling pathway [17,25]. Furthermore, AIB-1 facilitates the transition of downstream genes encoding cyclin D1 and the insulin-like growth factor-1 (IGF1) pathway [14,18,19], and it promotes the epithelial–mesenchymal transition through its interaction with ERα and worse outcomes in Erα-positive BCa [19,26]. In tamoxifen (TAM)-treated patients, high AIB-1 expression is associated with TAM resistance and poorer DFS [19,27,28,29]. The overexpression of AIB-1 correlated with poor prognosis in TNBC patients [19,30].

TIF-2 is frequently overexpressed in various neoplasms. Recurrent prostate cancers have exhibited high expression levels of THE androgen receptor, TIF-2, and SRC-1 [31]. TIF-2 correlates significantly with lymph node (LN)-positive BCa [32].

SRC-1 frequently correlates with high Her2/neu expression, LN metastasis, disease recurrence, poor DFS, and more advanced disease stage in BCa [33,34]. SRC-1 is a coactivator that can switch BCa from a steroid-responsive to a steroid-resistant state and promote the aggressive BCa phenotype. It has been implicated in aromatase inhibitor-resistant recurrent BCa [35]. SRC-1 and its homolog transcriptional co-activators p/CIP have been shown to be the coactivators for NF-kB, CREB, and STAT-1 [36].

NF-kB is a pleiotropic transcription factor and is the key activator of genes involved in host immunity and inflammatory responses with the induction of a large number of genes that influence cellular proliferation and inflammation. NF-kB activity promotes tumor proliferation, regulates cell apoptosis, and also induces the epithelial–mesenchymal transition, which facilitates distant metastasis and transactivates the expression of cyclin D1 and c-myc [37,38].

C-Jun is a component of the transcription factor AP-1. Extra- or intracellular signals, including growth factors and transforming oncoproteins, stimulate the phosphorylation of c-Jun at serine 63/73 and activate c-Jun-dependent transcription. Activated c-Jun has been demonstrated to be associated with proliferation and angiogenesis [39], as well as epithelial stem cell expansion [40].

Cyclin D1 is frequently overexpressed in BCa and contributes to ERα activation in BCa. AIB-1 and other steroid receptor coactivators can enhance the functional interaction of ERα with the cyclin D1 promoter [41], while cyclin D1 can recruit SRC-1 and AIB-1 to ERα in the absence of a ligand [42]. High cyclin D1 expression is associated with high proliferation and a higher risk of death from BCa in ERα-positive BCa. However, no significant prognostic impact of cyclin D1 expression has been found among ERα-negative cases [43], and the reverse relationship was demonstrated for cyclin D1 overexpression in invasive ductal carcinoma [44].

Overall, ERα-coactivator proteins enhance ligand-dependent and ligand-independent ERα signaling, progression, endocrine therapy resistance, and metastasis in BCa. Suen et al. [45] demonstrated that AIB-1 selectively enhances ERα but does not enhance ERβ-dependent gene transcription. TAM-induced AIB-1 recruitment to the ER-ERE enhanced interaction between AIB-1 and ERα but not ERβ. However, Liu et al. [46] observed opposing actions of ERα and ERβ with the dominance of ERβ over ERα in the activation of cyclin D1 gene expression. Estrogens, which activate cyclin D1 gene expression with ERα, inhibit expression with ERβ. The different recruitments of AIB-1 to ERα and ERβ may, in part, explain the different associations between ERs and response to endocrine treatment [47].

On the other hand, Bai et al. [48] observed that both ERα and ERβ can interact with the coactivator receptor interaction domains (RIDs) of all three SRC isoforms in living cells. Other studies have also demonstrated that ERβ transactivation recruits members of the SRC family [49,50]. The phosphorylation of AF-1 by MAP kinase (MAPK) leads to the recruitment of SRC-1 by ERβ and provides a molecular basis for the ligand-independent activation of ERβ via the MAPK cascade [51]. ERβ expression was significantly correlated with SRC-1, TIF-2, and NCOR protein levels in BCa and the upregulation of expression levels of ERβ and cofactors during the development of intraductal carcinomas [32]. ERβ and GRIP1/TIF2 has been shown to interact in vitro in a ligand-dependent manner and the transcriptional responses to estrogen in nonsmall cell lung cancer cells [52] and colon cancer via ERβ [53].

In summary, the combinations of ligand and ER subtypes can effectively recruit the three p160 coactivators albeit with differences in the levels and dose–response for coactivator recruitment by some of the ligands, with respect to their agonist activity [49,54]. Thus, coactivators seem to play an important role in directing ERβ-regulating genes or gene sets, further contributing to the functional complexity of ERβ.

Our previous study showed that high ERβ1 protein expression in BCa-associated myofibroblasts (MFs) was significantly associated with AIB-1 and TIF-2 expression in high-grade carcinoma with desmoplastic reaction and heavy lymphocytic infiltration [55]. Furthermore, our recent studies showed that high ERβ1 protein expression in ERα-negative BCa was correlated with high Ki-67, P53, and Her2/neu expression [9], and the expression of high ERβ2 and -5 isoform mRNAs is a poor risk factor and associated with high Ki-67 expression in BCa subtypes and subgroups [10]. As Erβ has strong affinity preferences for particular coactivators, in this study, we aimed to identify specific co-activators that interact with the Erβ isoform and are involved in the progression of BCa with ERβ expression.

## 2. Materials and Methods

### 2.1. Patients

All procedures involving patients with Bca were performed according to the ethical standards of the Institutional Research Board, Bridgeport Hospital, Bridgeport, CT (IRB# 090101). This study included 65 Erα-negative (43 TNBC) and 73 ERα-positive BCa from 138 patients with a follow-up period from 2003 to 2010. The demographic and clinical characteristics of all subjects were retrieved from medical records and cancer registry reports, as well as pathology records for hormone receptor reports, histologic types, tumor grades, tumor size, and AJCC tumor stages. Histological grades were assessed according to the Bloom–Richardson classification criteria. The AJCC tumor stages consisted of 75 in stage 1, 45 in stage II, and 18 in stage III. The follow-up period ranged from 1 to 96 months (median: 60 months); 20 patients died during this period. The phenotypic BCa patterns were determined according to Erα, HER2/neu, and progesterone receptor (PR) status following consensus guidelines. The proliferation marker Ki-67 was evaluated for all tumors. The molecular types comprised 50 luminal A (Erα^+^/PR^+^/HER2^−^), 25 luminal B (Erα^+^ and/or PR^+^/HER2^+^/Ki-67^+^), 17 HER2 (Erα^−^/PR^−^/HER2^+^), 17 basal-like (ERα^−^/PR^−^/HER2^−^/CK5/6^+^), and 29 unclassified types [10].

### 2.2. Tissue Microarray (TMA) Preparation

Hematoxylin and eosin sections of formalin-fixed paraffin-embedded (FFPE) tumor samples were evaluated. The TMA blocks were constructed using triplicate 0.6 mm diameter cores selected from the most representative tumor cellular areas of the primary Bca.

### 2.3. Immunohistochemistry

Standard immunohistochemistry (IHC) was performed using 4 µm thick sections of TMA slides of BCa following antigen retrieval with a steam-heating (95 °C) system in 0.01 M citrate buffer (pH 6.0) for 20 min or 1 mmol/L Tris–EDTA buffer at pH 9.0. The slides were stained with the appropriately diluted antibodies (Table 1) using an automated immunostainer (Dako, Santa Clara, CA, USA). Different clones of ERβ isoform antibodies, prognostic markers, and coactivators (Table 1) were tested for the optimum and reproducible immunoreaction, following the standard IHC testing protocol established in our laboratory. The IHC testing was conducted on the following antibodies: Erα, Erβ1, Erβ2, ERβ5, p-c-Jun (1:100), cyclin D1 (1:50), NF-kBp65 (1:100), SRC-1 (1:100), TIF-2 (1:50), AIB-1 (1:100), Ki-67, P53, CK 56, PR, and Her2/neu. The ERβ1 (38/AR385-10R), ERβ2 (57/3), and ERβ5 (5/75) antibody clones used in our previous study [10] and in this study have been tested by many investigators [7]; the immunogens were found to be peptide specific to the ERβ2 and Erβ5 splice variants [56,57,58,59,60,61]. Under the optimum immunostaining condition, ERβ1 (385p/AR385-10R) antibody displayed a consistent immunoreaction with each IHC test. Myofibroblasts were identified by smooth muscle actin staining using the EnVision G/2 double stain system. The positive and negative tissue and reagent controls were used. The immunoreactions of nuclear staining were evaluated using a semiquantitative Allred scoring system [10], summing the proportion of positive cells (scored on a scale of 0–5) and staining intensity (scored on a scale of 0–3) to produce a cumulative score of 8. A total score of 0–2 was regarded as negative, and a total score > 3 with 1–10% immunoreactive cells as positive. For Erβ isoform protein expression, >20% nuclear positivity was taken as the cutoff of positivity for ERβ1 and 2 isoforms, while >40% was applied for Erβ5 protein expression [10]. Over 1% of ERα and PR nuclear staining was considered positive. The Her2/neu expression was interpreted following the HercepTest kit guidelines and was scored according to the ASCO/CAP guidelines and considered positive for 3+ Her2/neu staining or 2+ Her2 staining with fluorescent in situ hybridization positivity. A nuclear immune reaction of Ki-67 > 15% and p53 > 5% was considered positive. The positive nuclear reaction of AIB-1, TIF-2, SRC-1, NF-kB, cyclin D1, and p-c-Jun in BCa were compared with those of normal breast tissues.

### 2.4. Statistical Analysis

The associations and correlations between the Erβ isoform protein, coactivators, and clinical characteristics were assessed for the entire cohort and the subtypes and subgroups of BCa using Fisher’s exact test and by Spearman’s rank-order test, respectively. Overall survival (OS) was calculated from the date of BCa diagnosis to death or the last follow-up visit, and the OS outcomes were estimated using Cox univariate and multivariate proportional hazard (PH) regression models. The hazard ratios were determined with 95% confidence intervals. Results with a *p*-value < 0.05 were considered significant.

This study sample size was sufficient statistically to detect correlations as small as ±0.17 and to detect relationships that explain at least 3% of the variance in dependent variables. All analyses were conducted using SAS 9.4 (SAS Institute Inc., Cary, NC, USA).

## 3. Results

The immunostaining of ERβ isoform 1, 2 and 5 proteins was strongly positive in the nuclei of luminal epithelial and myoepithelial cells, and stromal cells including fibroblasts, myofibroblast (MF), endothelial cells, and lymphocytes in the benign breast tissues, whereas that of Erα protein was positive only in the nuclei of luminal epithelial cells. The polyclonal ERβ1 (385p/AR385-10R) and ERβ5 (57/3) antibodies produced a stronger nuclear staining and some cytoplasmic staining than ERβ2. ERβ isoform 1, 2, or 5 protein expression was detected in 61.5%, 44.9%, and 59.5% of the entire cohort, respectively. ERβ1 protein expression showed differential expression in BCa subtypes with higher expression in well-differentiated duct carcinoma and lobular carcinoma than in poorly differentiated BCa. The ERβ1 protein expression was coexpressed with a high Her2/neu and p53 expression in the ERα-negative BCa. A. high Ki-67 positivity > 15% correlated with ERβ1, ERβ2, and/or ERβ5 protein expression in the various subtypes of BCa, as shown in our previous study [10].

**Figure 1:**
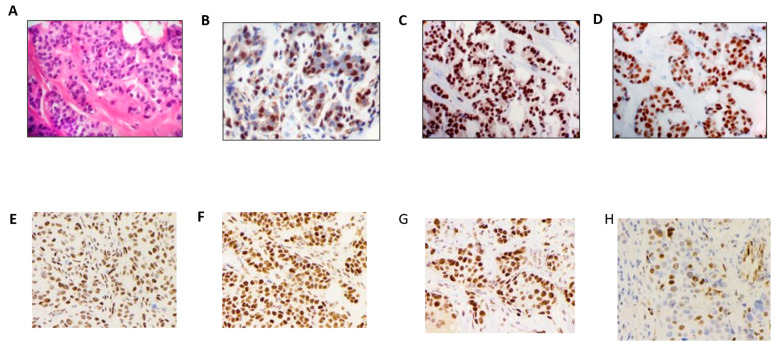
Immunohistochemistry stains of ERβ1 expression and coactivators in infiltrating duct carcinoma: (**A**) H & E staining of infiltrating duct carcinoma; (**B**) Erβ1 expression in the nuclei of benign epithelial cells and myoepithelial cells, stromal cells, and lymphocytes; (**C**) ERβ1 expression in the nuclei of neoplastic epithelial cells of infiltrating carcinoma and stromal cells and lymphocytes (immunohistochemistry staining using polyclonal ERβ1 385p/AR385-10R antibody); (**D**) ERα is expressed only in the nuclei of epithelial cells (original magnification 20×); immunohistochemistry stains of (**E**) AIB-1, (**F**) TIF-2, (**G**) SRC-1, and (**H**) p-c-Jun coactivators showing a positive nuclear reaction in the infiltrating carcinoma (original magnification 40×).

A high immunoreaction, as determined by an Allred score > 3, for AIB-1, TIF-2, SRC-1, NF-kB, and p-c-Jun protein expression was consistently observed in the nuclei of neoplastic epithelial cells, as well as in some stromal cells, particularly in MF (Figure 1). The nuclear expression of ERβ1 in epithelial cells was positively correlated with that in MF. On the contrary, ERα was neither expressed in the stromal cells nor in the MF. The ERβ1 expression was significantly associated with AIB-1, TIF-2, and p-c-Jun and with high-grade carcinoma with desmoplastic reaction and heavy lymphocytic infiltration. The nuclear expression of AIB-1, TIF-2, NF-kB, and p-c-Jun in MF gradually increased from the benign proliferative disease to carcinoma. Overall, AIB-1 protein expression was exclusively present in BCa and high-grade tumors and was higher in invasive BCa than in benign proliferative breast tissues. The cyclin D1 reaction levels in ERα-positive BCa (32.9%) were higher than those of ERα-negative BCa (11.4%) and TNBC (9.4%). Overall, the positive immunoreaction levels of cyclin D1 and p-c-Jun were lower than those of AIB-1, TIF-2, and NF-kB.

### 3.1. Association of Coactivators and Clinical Parameters in BCa Subtypes

A high cyclin D1 immunoreaction was positively associated with ERα-positive BCa, while that of TIF-2 and SRC-1 was associated with P53 > 5% positivity and that of p-c-Jun was associated with high Her2/neu expression (Table 2). However, there was an inverse association between cyclin D1 expression and luminal-B-type and TNBC (Table 3).

### 3.2. Spearman Rank Order Correlation between Coactivators and ERβ Isoforms

High expression levels of coactivators were significantly and differentially correlated with the expression of ERβ isoforms and clinical parameters. In the entire cohort (Table 4), high expression levels of AIB-1, TIF-2, and NF-kB were correlated with high ERβ1 and -5 expressions, while SRC-1, cyclin D1, and p-c-Jun were not associated with any of the ERβ isoforms. A high expression of ERβ5 isoform was correlated with high Ki-67, her2/neu, P53, and high-grade BCa; high ERβ1 expression was correlated with high Ki-67, high-grade and large-size BCa; and ERβ2 expression was correlated with lymph-node positive BCa and luminal-A-type BCa.

In the subtypes and subgroups of BCa (Table 5), the coexpression of high AIB-1, NF-kB, p-c-Jun, and TIF-2 and ERβ isoforms was significantly correlated with poor clinical prognostic markers, such as high Ki-67, p53, high-grade BCa, large-size BCa, and/or positive LN and with different types of BCa and molecular types. The coexpression of cyclin D1 and ERβ5 was correlated with ERα- and PR-positive BCa and luminal-A-type BCa, while p-c-Jun and ERβ5 were correlated with luminal-B-type BCa. Furthermore, the coexpression of high ERβ1 and NF-kB, as well as TIF-2 was correlated with high-grade BCa, and the expression of high ERβ1 and cyclin D1 was correlated with high Her2/neu BCa and luminal-B-type. Coexpression of TIF-2 and both of the ERβ5 and ERβ1 isoforms in TNBC suggests that TIF-2 may coregulate the proliferation and progression of Erβ-expressing TNBCs.

Among the ERβ isoforms, the ERβ 1 and -5 isoforms, predominantly ERβ5, were significantly correlated with coactivators in BCa, while ERβ2 was not associated with coactivators. Among the coactivators, AIB-1, NF-kB, p-c-Jun, and TIF-2 were significantly associated with ERβ isoform expression, while SRC-1 was not. Thus, SRC-1 seems independent of the other coactivators.

### 3.3. Cox Univariate OS and Cofactors Expression in BCa Subtypes and Subgroups

Using a Cox univariate proportional hazards model (Table 6), it was found that among the entire cohort, AIB-1, TIF-2, SRC-1, and NF-kB did not show any significant association with OS. However, in the subgroups, cyclin D1 expression was the risk factor for OS in ERα-positive BCa (*p* = 0.0336), PR-positive BCa (*p* = 0.0128), and luminal-A-type BCa (*p* = 0.0320).

## 4. Discussion

Studies on the role of coactivators in BCa have largely been investigated in ERα-positive BCa. In ERα-positive BCa, AIB-1 amplification has been associated with worse outcomes [26], progression of these tumors [62], resistance to TAM, early relapse during treatment, and distant recurrences. Moreover, high AIB1 expression in patients with Her2/neu-overexpressing tumors has been associated with an increased risk of relapse on tamoxifen [63] and, along with poor prognostic factors, with poorer DFS and OS in ERα-positive and -negative BCa [24]. This supports the notion of crosstalk between ERα and growth factor receptor pathways through specific coactivator proteins. Furthermore, high expression levels of cyclin D1 were significantly correlated to ERα positivity and with luminal A type [64], as well as high proliferation and a higher risk of death in ERα-positive BCa [43].

Studies on the role of ERβ isoforms and cofactors in BCa are limited. In this study, the most pertinent findings are the significant association and correlation between the expression of the Erβ5 and/or Erβ1 isoforms and AIB-1, NF-kB, TIF-2, p-c-Jun, and cyclin D1 coactivators in the BCa subtypes and subgroups. However, ERβ2 was not associated with coactivators and SRC-1 was not associated with ERβ expression. The coactivators were found to be differentially correlated with ERβ5 and/or ERβ1 expression in ERα-positive and ERα-negative BCa, as well as with TNBC and different molecular types of BCa. Their coexpression is associated and correlated with high-grade and large-sized tumors and high Her2/neu, p53, and Ki-67 positive BCa. High Ki-67 expression in BCa with high NF-kB, TIF-2, and AIB-1 expression suggests that the coactivators may be involved in the proliferation and growth of BCa. The coexpression of both ERβ5 and ERβ1 and TIF-2 in TNBC suggests that both the TIF-2 and ERβ isoforms may be implicated in poor prognosis in TNBC. The coexpression of high ERβ expression and AIB-1 and TIF-2 in MF in high-grade carcinoma with desmoplastic reaction and heavy lymphocytic infiltration suggests that the activation of AIB-1 and TIF-2 signal transductions in the MF may be involved in the initiation and progression of ERβ1-expressing BCa [65], as MF are the predominant cells in the cancer microenvironment that orchestrate the epithelial–mesenchymal crosstalk [66]. Tzelepi et al. [67] also reported that AIB-1 was more frequently expressed in the MF of dysplastic or cancer-associated mucosa stroma compared with normal mucosa. Enhanced nuclear ERβ1 expression and elevated nuclear AIB-1 expression were more frequently noted in the MF of carcinomas of an advanced stage, supporting the notion of the possible role of these coactivators in the initiation and progression of colorectal carcinomas through paracrine actions [22].

Although ERβ2 expression in this study was not associated with the coactivators, others have reported that ERβ2 mRNA levels are correlated with AIB-1 mRNA levels [68], and ERβ2 protein expression was found to be strongly associated with p-c-Jun and NF-kBp65 in ERα-negative BCa [69].

Furthermore, SRC-1 in our study was not correlated with any ERβ isoforms in BCa. However, others [70] have observed that patients with high expression levels of Her2/neu in combination with SRC-1 have a greater probability of recurrence on endocrine treatment compared with those who are Her2/neu positive but SRC-1 negative. SRC-1 was associated with nodal positivity and resistance to endocrine treatment. Fleming et al. [34] reported that SRC-1 was inversely associated with ERβ, negatively associated with DFS, and positively correlated with Her2/neu.

There was no significant association between OS and AIB-1, TIF-2, SRC-1, and NF-kB. However, among the subtypes, cyclin D1 was a significant risk factor for OS in ERα-positive BCa (*p* = 0.0336), PR-positive BCa (*p* = 0.0128), and luminal-A-type BCa (*p* = 0.0320). Others reported that among the ERα-negative subgroup, strong AIB-1 protein expression correlated with poorer DFS and overall survival and correlated with the amplification of the Her2/neu gene [24]. AIB-1 was found to enhance the estrogen-dependent induction of cyclin D1 expression by ERα [41].

## 5. Conclusions

Our study is the first comprehensive simultaneous investigation of the correlation and association of the ERβ1, ERβ2, and ERβ5 isoforms with multiple coactivators, including AIB-1, NF-kB, cyclin D1, SRC-1, p-c-Jun, and TIF-2, in the entire cohort, as well as in the subtypes and subgroups of BCa. AIB-1, NF-kB, p-c-Jun, and TIF-2 were found to be associated and correlated with ERβ5 and ERβ1 expression, as well as with poor clinical parameters, and were differently associated with the subtypes of BCa, including different molecular types. ERβ5 was determined to be the predominant ERβ isoform associated and correlated with coactivators in the subtypes and subgroups of BCa, while ERβ2 did not demonstrate the relationship. High Ki-67 expression with the coexpression of coactivators and ERβ5 suggests a potential involvement of the coactivators in the proliferation of ERβ-expressing BCa. SRC-1 is not associated with any ERβ expression. Cyclin D1 was the risk factor for OS only in the BCa subtypes.

In summary, although this study was limited by its relatively small sample size with respect to the subtypes and groups, we firmly believe that the sample size sufficiently supported both our positive and negative results.

ERβ interacts with the members of the SRC family and other coactivators and coregulate the development and growth of BCa [49,50,51,54]. As ERβ isoforms were found to be the risk factors and associated with unfavorable clinical outcomes in BCa in our previous study [10], the significant correlation between ERβ isoforms and the coactivators in the present study supports the notion that the coactivators are co-implicated in the proliferation of BCa and the risk factors of ERβ-expressing BCa.

Previous studies [71,72,73,74] have demonstrated that the activity of ERs depends on the coordinated activity of ligand binding, PTM, and interaction with their partner coregulators and that distinct receptor subtype-specific coregulators are recruited at the transcription sites and factors, such as ERα or ERβ. Thus, further studies with other coregulators and large cohorts of BCa subgroups and subtypes, including the BRCA-1-associated TNBC [75], are needed to determine the involvement of specific coactivators in ERβ-expressing BCa.

This may provide insights into the potential usefulness of the coactivators as therapeutic targets in BCa in the adjuvant setting. The blocking of coactivators may slow disease progression and potentially play an important role in the adjuvant setting to prevent disease recurrence and the development of metastases in the subtypes of BCa [16,37,38,76,77,78].

## Figures and Tables

**Table 1:** Antibodies for immunohistochemistry study.

Antibody	Antibody Clone	Supplier
ERβ1	385P/AR 385-10R	Biogenex, San Ramon, CA, USA
ERβ2	MCA2279S/57/3	Bio-rads, Hercules, CA, USA
ERβ5	MCA4676/5/25	Bio-rads, Hercules, CA, USA
AIB-1	clone 34, mouse monoclonal	BD Transductuction Labs, San Jose, CA, USA
TIF-2	clone 29, mouse monoclonal	BD Transductuction Labs, San Jose, CA, USA
NF-kB p65	ABCAM E379	Waltham, MA, USA
SRC-1	clone 128E7, rabbit monoclonal	Cell Signaling Technology, Daners, MA, USA
Cyclin D1	DCS-6	DAKO, Carpintena, CA, USA
p-c-Jun	822, KM-1	Santa Cruz Biotech, Dallas, TX, USA
Actin-SMA	clone 2A4, mouse antihuman	DAKO, Carpintena, CA, USA
Ki-67	MIB-1	DAKO, Carpintena, CA, USA
P53	D07	DAKO, Carpintena, CA, USA
HER2/neu	HerceptTest	DAKO, Carpintena, CA, USA
ERα	ID5	DAKO, Carpintena, CA, USA
PR	Pg363	DAKO, Carpintena, CA, USA

**Table 2:** Association between coactivators and clinical characteristics.

		AIB-1			NF-kB			TIF-2			SRC-1			p-c-Jun			Cyclin D1		
Variables		Pos	Neg	*p*-Value *	Pos	Neg	*p*-Value	Pos	Neg	*p*-Value	Pos	Neg	*p*-Value	Pos	Neg	*p*-Value	Pos	Neg	*p*-Value
**ERα status**	Pos	49	11	0.3	58	5	1	60	4	1	49	16	1	48	14	0.83	22	50	**0.024**
	neg	49	6		43	3		48	3		43	13		40	14		7	57	
**Her-2/neu**	Pos	34	6	1	35	3	1	33	1	0.67	29	11	0.49	33	4	**0.022**	14	29	0.08
	Neg	69	11		66	5		75	6		63	17		55	24		17	78	
**PR**	Pos	46	9	0.79	51	4	1	54	4	1	47	13	0.83	43	14	1	20	47	0.07
	Neg	52	8		50	4		54	3		45	15		45	14		11	60	
**Ki-67**	>15%	27	1	**0.07**	25	1	0.67	27	1	1	70	5	0.61	23	8	0.61	8	21	0.46
	<15%	71	16		76	6		81	6		22	23		68	20		23	86	
**Grade**	Grade 2/3	85	16	0.69	88	7	1	93	6	1	80	23	0.54	79	22	0.19	29	91	0.36
	Grade 1	13	1		13	1		15	1		12	5		9	6		22	16	
**Tumor size**	>2 cm	42	7	1	43	3	1	44	2	0.7	39	11	0.83	36	12	1	15	39	0.29
	<2 cm	56	10		58	5		64	5		53	17		52	16		16	68	
**Nodal status**	Pos	23	3	0.76	26	1	0.67	25	2	0.67	22	7	1	20	6	1	7	24	1
	Neg	75	14		76	7		83	5		70	21		68	22		24	83	
**CK5/6**	Pos	12	2	1	13	1	1	14	1	1	13	1	0.18	11	24	0.75	3	13	1
	Neg	86	15		88	7		84	6		79	27		77	24		28	94	
**P53 > 5%**	Pos	60	6	0.3	50	2	0.27	56	0	**0.013**	48	8	**0.032**	46	10	0.13	14	44	0.68
	Neg	48	11		51	6		52	7		44	20		42	18		17	63	

* All *p*-values were calculated with the Fisher’s Exact test.; bold: significant *p*-value < 0.05.

**Table 3:** Associations between coactivators and molecular types of breast cancers.

	AIB-1			NF-kB			TIF-2			SRC-1			p-c-Jun			cyclin D1		
	Pos	Neg	*p*-Value *	Pos	Neg	*p*-Value	Pos	Neg	*p*-Value	Pos	Neg	*p*-Value	Pos	Neg	*p*-Value	Pos	Neg	*p*-Value
**Types**																		
**Luminal A type (50)**	31	8	0.26	36	5	1	40	4	0.42	35	7	0.66	30	12	0.49	14	36	0.29
	67	9		69	3		69	3		57	19		58	16		17	71	
**Luminal B type (25)**	21	3	1	23	2	1	21	0	0.34	17	7	0.43	19	3	0.27	10	16	**0.039**
	77	14		78	6		87	7		95	21		9	25		21	91	
**Basal-like type (17)**	13	2	1	14	1	1	5	1	1	13	3	0.51	11	5	0.53	2	15	0.36
	85	15		87	7		2	6		79	26		77	23		29	92	
**HER2 type (17)**	12	3	0.46	14	1	1	15	0	0.59	14	1	0.19	11	4	0.75	5	12	0.53
	86	14		87	7		93	7		78	27		77	24		26	95	
**TNBC (43)**	32	3	0.26	29	2	1	34	2	1	28	7	0.64	25	11	0.34	4	40	**0.009**
	66	14		72	6		74	5		64	21		63	17		27	67	

* All *p*-values calculated with the Fischer Exact test.; bold: significant *p*-value < 0.05.

**Table 4:** Spearman rank correlation of ERβ isoform protein expression with coactivators and clinical parameters in the entire cohort.

	ERβ1 Protein	ERβ2 Protein	ERβ5 Protein
**AIB-1**	**0.19 (0.047)**	0.066 (0.48)	**0.25(0.0064)**
**NF-kB**	**0.21 (0.028)**	0.13 (0.17)	**0.41 (<0.0001)**
**TIF-2**	0.24 (0. 008)	−0.01 (0.90)	**0.31 (0.0005)**
**SRC-1**	0.17 (0.07)	0.07 (0.45)	0.12 (0.17)
**p-c-Jun**	−0.04 (0.63)	0.08 (0.38)	0.06 (0.49)
**Cyclin D1**	0.11 (0.18)	0.11 (0.18)	0.13 (0.14)
**Ki-67**	**0.38 (<0.0001)**	0.14 (0.088)	**0.34 (<0.0001)**
**P53**	0.085 (0.33)	0.06 (0.49)	**0.30 (0.029)**
**Grade 3**	**0.17 (0.049)**	0.17 (0.071)	**0.19 (0.024)**
**>2 cm**	**0.17 (0.04)**	0.06 (0.49)	0.10 (0.23)
**Her2/neu+**	0.26 (0.89)	0.25 (0.10)	**0.31 (0.045**)
**LN+**	0.09 (0.29)	**0.23 (0.007)**	0.14 (0.08)
**ERα+**	0.01 (0.81)	0.15 (0.07)	−0.0005 (0.99)
**PR+**	0.005 (0.95)	0.07 (0.39)	−0.08 (0.32)
**Luminal A type**	0.005 (0.94)	**0.17 (0.045)**	−0.08 (0.32)
**Luminal B type**	−0.01 (0.87)	−0.05 (0.54)	−0.07 (0.37)
**HER2 type**	−0.07 (0.39)	−0.09 (0.27)	0.08 (0.36)
**Basal type**	0.03 (0.7)	0.05 (0.58)	**0.17 (0.038)**

Bold: significant *p*-value < 0.05.

**Table 5:** Spearman rank correlation of ERβ isoforms and coactivators in subtypes and subgroups.

	ERβ 5 Expression		ERβ1 Expression	ERβ2 Expression
Correlation with	subgroups	r (*p*-value) *	r (*p*-value)	r (*p*-value)
**AIB-1**	ER**α+**	0.34 (0.0082)	0.18 (0.16)	0.15 (0.25)
	ERα-	0.18 (0.20)	0.21 (0.11)	0.012 (0.93)
	Luminal A type	0.38 (0.019)	0.22 (0.17)	0.15 (0.34)
	HER2 type	0.54 (0.035)	0.46 (0.08)	−0.09 (0.73)
	>2 cm tumor	0.29 (0.042)	0.11 (0.44)	−0.16 (0.27)
	Grade 3	0.23 (0.02)	0.14 (0.15)	−0.12 (0.21)
	Her2/neu+	0.35 (0.027)	0.19 (0.23)	0.1 (0.34)
**NF-kB**	ERα+	0.43 (0.0004)	0.22 (0.07)	0.24 (0.06)
	ERα-	0.39 (0.0078)	0.20 (0.17)	0.05 (0.72)
	Luminal A type	0.51 (0.0007)	0.15 (0.33)	0.11 (0.50)
	HER2 type	0.68 (0.035)	0.07 (0.08)	−0.03 (0.9)
	Ki-67 > 15%	0.31 (0.019)	0.16 (0.23)	−0.07 (0.59)
	Her2/neu+	0.45 (0.0046)	0.20 (0.21)	0.15 (0.36)
	>2 cm tumor	0.29 (0.042)	0.14 (0.35)	−013 (0.39)
	Grade 3	0.42 (<0.0001)	0. 25 (0.013)	0.15 (0.16)
	PR	0.47 (0.0003)	0.023 (0.08)	−0.13 (0.33)
	LN+	0.47 (0.013)	−0.13 (0.51)	0.27 (0.16)
**TIF-2**	ERα+	0.35 (0.0039)	−0.21 (0.09)	−0.15 (0.23)
	ERα-	0.34 (0.043)	−0.13 (0.34)	−0.19 (0.16)
	TNBC	0.33 (0.046)	0.33 (0.05)	−0.19 (0.26)
	Luminal A type	0.35 (0.019)	0.17 (0.24)	0.28 (0.07)
	Ki-67 > 15%	0.32 (0.01)	0.15 (0.21)	−0.12 (0.33)
	p53 > 5%	0.28 (0.029)	0.12 (0.33)	0.12 (0.38)
	Grade 3	0.30 (0.0023)	0.25 (0.012)	0.05 (0.61)
	PR	0.28 (0.03)	0.16 (0.23)	0.19 (0.16)
**Cyclin D1**	ERα+	0.29 (0.011)	0.19 (0.11)	0.16 (0.18)
	Her2/neu+	0.09 (0.53)	0.3 (0.049)	0.12 (0.45)
	Luminal A type	0.25 (0.004)	0.11 (0.45)	0.25 (0.07)
	Luminal B type	0.1 (0.49)	0.45 (0.02)	0.02 (0.94)
	PR	0.24 (0.046)	0.22 (0.07)	0.09 (0.43)
**p-c-Jun**	Luminal B type	0.44 (0.039)	0.007 (0.97)	−0.008 (0.71)
**SRC-1**	ERα+	0.18 (0.17)	0.09 (0.49)	0.01 (0.93)
	ERα-	0.08 (0.48)	0.22 (0.07)	0.08 (0.52)
	Her2/neu+	0.53 (0.34)	0.10 (0.53)	−0.09 (0.54)
	PR	0.12 (0.34)	0.20 (0.11)	0.19/0.13

* bold: significant *p*-value < 0.05.

**Table 6:** Cox univariate overall survival analysis of coactivators in breast cancer subtypes and subgroups.

Subgroups (Case#)	AIB-1		NF-kB		TIF-2		SRC-1		p-c-Jun		Cyclin D1	
	*p*-Value	HR (CI)	*p*-Value	HR (CI)	*p*-Value	HR (CI)	*p*-Value	HR (CI)	*p*-Value	HR (CI)	*p*-Value	HR (CI)
ERα- positive BCa (73)	0.25	0.99 (0.96–1.011)	0.58	0.733 (0.25–2.17)	0.103	0.98 (0.96–1.004)	0.53	0.99 (0.97–1.02)	0.64	0.94 (0.77–1.02)	0.034	1.02 (1.001–1.037)
ERα -negative BCa (65)	0.23	1.01 (0.99–1.03)	0.76	1.1 (0.52–2.45)	0.22	1.01 (0.99–1.03)	0.79	0.99 (0.98–1.02)	0.24	1.01 (0.99–1.03)	0.68	0.99 (0.91–1.06)
TNBC (43)	0.5	1.008 (0.98–1.03)	0.54	0.71 (0.23–2.2)	0.17	1.02 (0.99–1.05)	0.43	0.99 (0.97–1.02)	0.49	1.008 (0.99–1.03)	0.99	0.06 (0.000–1.0000)
Her2/neu+ (39)	0.35	0.98 (0.97–1.013)	0.72	1.2 (0.44–3.29)	0.67	0.99 (0.97–1.022)	0.76	0.99 (0.96–1.03)	0.79	1.004 (0.98–1.03)	0.59	1.006 (0.98- 1.03)
PR+ (54)	0.54	0.99 (0.97–1.02)	0.59	0.73 (0.23–2.34	0.19	0.98 (0.96–1.007)	0.53	0.99 (0.97–1.016)	0.66	0.99 (0.97–1.021)	0.0128	1.03 (1.005–1.05)
Luminal A type (50)	0.87	1.003 (0.97–1.04)	0.97	1.04 (0.206–204)	0.1	0.9 (0.94–1.006)	0.72	0.99 (0.97–1.024)	0.59	0.99 (0.959–1.03)	0.032	1.028 (1.002–1.055)
Luminal B type (25)	0.12	0.95 (0.89–1.01)	0.48	0.56 (0.12–2.71)	0.34	1.1 (0.8–1.41)	0.61	0.99 (0.95–1.03)	0.87	1.004 (0.96–1.05)	0.61	1.007 (0.98–1.034)
HER2 type (17)	**		**		**		**		**		**	
Basal type (17)	0.12	1.03 (0.99–1.07)	0.21	3.1 (0.54–17.38)	0.34	1.03 (0.97–1.097)	0.93	1.001 (0.97–1.03)	0.96	1.001 (0.97–1.03)	0.99	0.46 (0.000–3.87)
Grade 2/3 (115)	0.97	1.000 (0.98–1.013)	0.93	0.97 (0.49–1.9)	0.78	0.99 (0.99–1.011)	0.2	0.99 (0.97–1.006)	0.71	1.003 (0.99–1.012)	0.23	1.009 (0.99–1.023)
Grade 1 (23)	**		**		**		**		**		**	
>2 cm tumor (51)	0.91	1.001 (0.98–1.02)	0.88	1.05 (0.54–2.05)	0.72	0.99 (0.98–1.013)	0.99	1.000 (0.98–1.02)	0.56	1.005 (0.98–1.023)	0.65	1.004 (0.99–1.02)
<2 cm tumor (87)	0.82	1.004 (0.97–1.03)	0.84	0.85 (0.18–3.9)	0.77	0.99 (0.97–1.02)	0.25	0.98 (0.94–1.02)	0.58	1.009 (0.978–1.04)	0.31	1.019 (0.98–1.04)
>15% Ki-67 (63)	0.85	0.99 (0.98–1.014)	0.9	1.05 (0.50–2.19)	0.27	0.99 (0.98–1.006)	0.19	0.99 (0.97–1.007	0.73	1.003 (0.98–1.020)	0.11	1.013 (0.99–1.03)
LN positive (34)	0.83	1.002 (0.98–1.03)	0.85	1.16 (0.25–5.33)	0.82	0.99 (0.98–1.02)	0.83	1.00 (0.9–1.02)	0.25	1.02 (0.99–1.06)	0.28	1.011 (0.99–1.03)
p53 > 5% (57)	0.59	0.99 (0.98–1.013)	0.92	0.95 (0.37–2.5)	0.097	0.98 (0.97–1.003)	0.31	0.99 (0.96–1.013)	0.31	1.011 (0.9–1.032)	0.57	0.99 (0.9–1.02)

** sample too small to reliably calculate COX; HR: Hazard Ratio, CI: Confidence Interval.

## Data Availability

The datasets generated during and/or analyzed during the current study are included in this manuscript. Any additional request is available from the corresponding author upon reasonable request.
